# Vaccination with nanoparticles combined with micro-adjuvants protects against cancer

**DOI:** 10.1186/s40425-019-0587-z

**Published:** 2019-04-26

**Authors:** Mona O. Mohsen, Matthew D. Heath, Gustavo Cabral-Miranda, Cyrill Lipp, Andris Zeltins, Marcos Sande, Jens V. Stein, Carsten Riether, Elisa Roesti, Lisha Zha, Paul Engroff, Aadil El-Turabi, Thomas M. Kundig, Monique Vogel, Murray A. Skinner, Daniel E. Speiser, Alexander Knuth, Matthias F. Kramer, Martin F. Bachmann

**Affiliations:** 1 Jenner Institute, Nuffield Department of Medicine, University of Oxford, Oxford, UK; 2 Department of BioMedical Research, Immunology RIA, Inselspital, University of Bern, Bern, Switzerland; 3 Bencard Adjuvant Systems, Dominion Way, Worthing, UK; 4 Latvian Biomedical Research & Study Centre, Riga, Latvia; 5 Institute of anatomy, University of Bern, Bern, Switzerland; 6 Theodor Kocher Institute, University of Bern, Bern, Switzerland; 7 Department of Medical Oncology, Bern University Hospital, University of Bern, Bern, Switzerland; 8 International Immunology Center, Anhui Agricultural University, Hefei, Anhui China; 9 Department of dermatology, University of Zurich, Bern, Switzerland; 10 Department of Oncology, University of Lausanne, Bern, Switzerland; 11 National Center for Cancer Care & Research (NCCCR), Doha, State of Qatar

**Keywords:** Cucumber-mosaic virus CuMV, Virus-like particle VLP, Microcrystalline tyrosine MCT, Nano-vaccine

## Abstract

**Background:**

Induction of strong T cell responses, in particular cytotoxic T cells, is a key for the generation of efficacious therapeutic cancer vaccines which yet, remains a major challenge for the vaccine developing world. Here we demonstrate that it is possible to harness the physiological properties of the lymphatic system to optimize the induction of a protective T cell response. Indeed, the lymphatic system sharply distinguishes between nanoscale and microscale particles. The former reaches the fenestrated lymphatic system via diffusion, while the latter either need to be transported by dendritic cells or form a local depot.

**Methods:**

Our previously developed cucumber-mosaic virus-derived nanoparticles termed (CuMV_TT_-VLPs) incorporating a universal Tetanus toxoid epitope TT830–843 were assessed for their draining kinetics using stereomicroscopic imaging. A nano-vaccine has been generated by coupling p33 epitope as a model antigen to CuMV_TT_-VLPs using bio-orthogonal Cu-free click chemistry. The CuMV_TT_-p33 nano-sized vaccine has been next formulated with the micron-sized microcrystalline tyrosine (MCT) adjuvant and the formed depot effect was studied using confocal microscopy and trafficking experiments. The immunogenicity of the nanoparticles combined with the micron-sized adjuvant was next assessed in an aggressive transplanted murine melanoma model. The obtained results were compared to other commonly used adjuvants such as B type CpGs and Alum.

**Results:**

Our results showed that CuMV_TT_-VLPs can efficiently and rapidly drain into the lymphatic system due to their nano-size of ~ 30 nm. However, formulating the nanoparticles with the micron-sized MCT adjuvant of ~ 5 μM resulted in a local depot for the nanoparticles and a longer exposure time for the immune system. The preclinical nano-vaccine CuMV_TT_-p33 formulated with the micron-sized MCT adjuvant has enhanced the specific T cell response in the stringent B16F10p33 murine melanoma model. Furthermore, the micron-sized MCT adjuvant was as potent as B type CpGs and clearly superior to the commonly used Alum adjuvant when total CD8^+^, specific p33 T cell response or tumour protection were assessed.

**Conclusion:**

The combination of nano- and micro-particles may optimally harness the physiological properties of the lymphatic system. Since the nanoparticles are well defined virus-like particles and the micron-sized adjuvant MCT has been used for decades in allergen-specific desensitization, this approach may readily be translated to the clinic.

**Electronic supplementary material:**

The online version of this article (10.1186/s40425-019-0587-z) contains supplementary material, which is available to authorized users.

## Introduction

Nanoparticles, specifically virus-like particles (VLPs), have succeeded as prophylactic vaccines and are now widely used. However, mounting an efficient immune response by therapeutic cancer vaccines is still a challenging area, highlighting the need for improved vaccine formulations. Three main parameters are pivotal for the development of an effective cancer vaccine: a cancer antigen, a delivery platform and an adjuvant. A large number of different formulations have been studied extensively in the past years; however head-to-head comparative data are still scarce and remain untested in humans [1]. Furthermore, vaccine formulations have not been studied or optimized with respect to the size of delivery platforms versus adjuvants.

Cucumber-mosaic virus (CuMV) coat protein may be expressed as a recombinant plant nano-sized virus-like particle (VLP) and has been studied as a promising candidate vaccine platform by displaying relevant epitopes for the induction of immune responses [2]. CuMV-VLPs are icosahedral nanoparticles capable of inducing both humoral and cellular immune responses by generating neutralizing antibodies (Abs), CD4^+^ T_H_ cells and CD8^+^ cytotoxic T lymphocytes (CTLs) [3, 4]. Previously, we have developed an engineered nano-sized cucumber-mosaic virus-derived VLP by incorporating a universal Tetanus toxoid epitope TT830–843 which we termed CuMV_TT_-VLPs [5]. The incorporation of the universal T cell epitope has been shown to be a powerful enhancer of the immune response in Tetanus toxoid immunized mice. Using the engineered nano-sized CuMV_TT_-VLPs as a vaccine platform in humans is also expected to enhance their immune responses, since this epitope is recognized in essentially all humans who all have memory CD4^+^ T cells specific for the epitope due to vaccination against tetanus [5]. We have shown in several studies that CuMV_TT_-VLPs nano-vaccine induce protective and therapeutic Ab responses in mice, horses and dogs [5–7].

Depot-forming adjuvants can prolong antigen presentation time to antigen-presenting cells (APCs), protect antigen from degradation and are optimal for T cell enhancement and clonal expansion [8, 9]. Microcrystalline tyrosine (MCT) is a classical adjuvant used in the niche area of allergy immunotherapy, some of which include products that are licensed or are currently in late stage clinical development [10]. MCT forms crystals of natural L-Tyrosine and due to their micron-size, they cannot readily enter the lymphatics and remain at the injection site, forming a depot and local inflammation [11]. The introduction of MCT as a depot was based on its favorable biodegradable properties where a 48 h half-life at the injection site has been previously reported, making it suitable to adapt within formulations designed for weekly-dose administration as is also often used for cancer vaccines [11–14]. Moreover, early immunological studies in various animal models showed a more favorable T_H_1-biased immunological profile compared with the widely used Alum adjuvant [12, 15]. This is also further indicated where immunological synergy is observed when MCT is combined with Monophosphoryl Lipid A®, since it was shown that a combination of MCT and MPL was synergistic in enhancing murine antigen specific IgG Ab responses without increasing antigen specific IgE responses [15]. A short-course allergy vaccine using this adjuvant combination has recently completed a successful Phase II study in Europe and has been marketed as a named patient product for a number of years [16]. Further evidence has now emerged on MCT’s more specific immunomodulatory functions. MCT activates the inflammasome in vitro and has been demonstrated to induce sustained and robust innate responses, including, specific adaptive T cell responses in a variety of immune-applications [11, 13, 14]. Consequently, proof of concept studies using MCT across a broader vaccine scope is being pursued more rigorously and has since highlighted its adaptable nature within various formulation designs with other adjuvants / delivery systems in optimizing immune responses [17].

Here we harnessed the influence of particle’s size on draining properties and their efficacy in producing effective CTLs response against an aggressive B16F10 tumour model by combining a CuMV_TT_ nano-vaccine with the micron-size MCT adjuvant. We compared the response to other commonly used adjuvants such as the well-established Alum and the potent B type immunostimulatory CpGs. We show that the micron-sized MCT is a powerful adjuvant for CuMV_TT_-p33 nano-vaccine displaying p33 epitope derived from LCMV, rivaling CpGs and clearly performing better than Alum in inducing CTLs and tumour protection. Thus, this combination may be an optimal way to formulate cancer nano-vaccines with micron-sized adjuvants thereby taking advantage of the physiological properties of the lymphatic system.

## Materials and methods

### Expression and production of CuMV_TT_-VLPs

CuMV_TT_-VLPs expression and production was performed as described in detail in [5].

### Electron microscopy

Physical stability and integrity of CuMV_TT_-VLPs were visualized by transmission electron microscopy using the Philips CM12 EM. For imaging, sample-grids were glow discharged and 5 μl of VLP solution was added for 30 s. The grids were then washed 3x with ddH_2_O and negativly stained with 5 μl of 5% uranyl acetate for 30 s. Finally, excess uranyl acetate was removed by pipetting and the grids were air dried for 10 min. Images were taken with 84,000x and 110,000x magnification.

### Mice

Wild type C57BL/6 mice were purchased from Harlan. *RAG2*^*−/−*^ mice on a C57BL/6 background were provided by Ochsenbein’ lab and were bred in our pathogen-free animal facility. All in vivo experiments used 8–12-week-old female. All animal procedures were performed in accordance with the Swiss Animals Act (455.109.1) (September 2008, 5th) of University of Bern.

### Stereomicroscopic imaging

WT C57BL/6 mice (8–12 weeks; Harlan) were anesthetized and prepared for imaging by shaving their right leg. Skin and adipose tissues were removed to expose the popliteal lymph node (LN) as described in detail in [18]. The anesthetized mice were then stabilized on a customized platform for imaging. The popliteal LN was located by bright field illumination imaging. A dose of 10 μg of CuMV_TT_-VLPs vaccine was labelled with Alexa Fluor 488 (AF488) according to the manufacturer’s instructions (Thermo Fisher SCIENTIFIC) and injected subcutaneously (s.c.) into the mouse footpad to study the draining kinetics of CuMV_TT_-VLPs. Fluorescent light illumination with a CCD Nikon camera was used for imaging.

### Development of CuMV_TT_-p33 nano-vaccine using bio-orthogonal cu-free click chemistry

CuMV_TT_-VLPs were derivatized using 10-fold molar excess of DBCO cross-linker (Dibenzocyclooctyne-*N*-hydroxysuccinimidyl ester) (Sigma-Aldrich) in 2 mM EDTA and 20 mM NaP, pH 7.5 for 30 min at RT in a shaker at 400 rpm. Excess uncoupled DBCO was removed by diafiltration steps. Modified p33 peptide H-KAVYNFATMGGCK(N3)-NH2 was purchased from (Pepscan PRESTO) and reconstituted using DMSO. 10-fold molar excess of the modified peptide was then added to the derivatized CuMV_TT_-VLPs. 5-fold molar excess of TCEP was added to liberate cysteine residues at the C-terminus on the VLPs. The coupling was performed for 1 h at RT in a shaker at 400 rpm. Excess peptide was removed using 100 kDa MWCO amicon centrifuge tubes (Sigma Aldrich). The efficiency of the coupling was tested by SDS-PAGE (Bio-RAD) and assessed by densitometric analysis of SDS-PAGE of CuMV_TT_-VLP monomer bands compared to CuMV_TT_-VLP monomer plus p33 after coupling.

### Depot effect with confocal microscopy

10 μg of AF488 CuMV_TT_-p33 nano-vaccine formulated or not formulated with 50 μl of 4% MCT adjuvant was injected in WT C57BL/6 mice footpads (8–12 weeks; Harlan) using isoflurane anesthesia. Popliteal LNs were collected 3 h, 24 h, 48 h, 96 h and 216 h after injection. Lymph nodes were fixed in PFA 2% for 3 h at RT. The LNs were transferred to CUBIC 1 medium [19] for 8 days at 37 °C and scanned at Leica SP8 with 10x lense, 1024 × 1024 resolution, tile scan was performed in LNs that were not fitting in the field of view of the 10x lense. Images were analyzed and segmented the individual CMV particles using Imaris Software v9.2.1 (Bitplane).

### Depot effect with trafficking experiment

10 μg of AF488 CuMV_TT_-p33 nano-vaccine formulated or not formulated with 50 μl of 4% MCT adjuvant was injected in WT C57BL/6 mice footpads (8–12 weeks; Harlan) using isoflurane anesthesia. Popliteal LNs were collected 3 h, 24 h, 48 h, 96 h and 216 h after injection and treated with collagenase D (Roch) in 10%FSC containing DMEM for 25 min at 37 °C. Cells were stained with live/dead dye (eBioscience) and analyzed for total number of FITC^+^ (CuMV_TT_-p33). Naïve mice were used as a control.

### Measuring p33 specific CD8^+^ T cell response in the spleen

Six groups of WT C57BL/6 mice (8–12 weeks old; Harlan) were vaccinated s.c. with a single dose of: 1st group 70 μg of CuMV_TT_-VLPs, 2nd group 70 μg of CuMV_TT_-Actin nano-vaccine, 3rd group 70 μg of CuMV_TT-_p33 nano-vaccine_,_ 4th group 70 μg of CuMV_TT-_p33 nano-vaccine admixed with 15 nmol of B type CpGs 5′′-TCC ATG ACG TTC CTG ATG CT-3′′) (20 mer) (Invivogen), 5th group 70 μg of CuMV_TT-_p33 nano-vaccine formulated with 4% MCT adjuvant (40 mg/ ml) (Allergy Therapeutics Ltd. Worthing, UK) and the 6th group 70 μg of CuMV_TT-_p33 nano-vaccine formulated with 100 μl of Alhydrogel adjuvant 2% (InvivoGen). Formulating CuMV_TT_-p33 with MCT or Alum requires prolonged mixing of both components for 1 h at RT in shaker at 400 rpm to ensure adequate adsorption of the VLPs on MCT or Alum surface. Seven days later, spleens were collected and staining was performed using Fc-block, live/dead, anti-CD8 (eBioscience) and p33 (KAVYNFATM) tetramer designed using H-2Db allele and PE fluorochrome (TCMetrix).

### Intra-cellular cytokine (ICS) staining for IFN-γ and TNF-α

Intra-cellular cytokine staining was performed on spleens and TILs of vaccinated WT C57BL/6 mice for measuring of IFN-γ and TNF-α cytokines as described in detail in [20].

### Tumour experiments

1 × 10^6^ cells of B16F10p33 melanoma cell line (From Ochsenbein lab) was injected into the flank of *RAG2*^*−/−*^ C57BL/6 mice (From Ochsenbein lab). Twelve days later the growing tumours were collected and processed into ~2mm^2^ fragments for transplantation into the flank of WT C57BL/6 mice (8–12 weeks old; Harlan) under full anesthesia. The transplanted WT C57BL/6 mice were treated 3 times over 14 days (mice in the control group reached the humane end-point at day 14). In the first tumor experiment mice were vaccinated s.c. as follows: 1st group 70 μg of CuMV_TT_-VLPs, 2nd group 70 μg of CuMV_TT_-p33 nano-vaccine and 3rd group 70 μg of CuMV_TT_-p33 nano-vaccine formulated with 4% MCT adjuvant (40 mg/ ml) (Allergy Therapeutics Ltd. Worthing, UK). In the second tumour experiment mice were vaccinated s.c. as follows: 1st group 70 μg of CuMV_TT_-VLPs, 2nd group 70 μg of CuMV_TT_-p33 nano-vaccine admixed with 15 nmol of B type CpGs 5′′-TCC ATG ACG TTC CTG ATG CT-3′′) (20 mer) (Invivogen), 3rd group 70 μg of CuMV_TT_-p33 nano-vaccine formulated with 4% MCT adjuvant (40 mg/ ml) (Allergy Therapeutics Ltd. Worthing, UK) and 4th group 70 μg of CuMV_TT_-p33 nano-vaccine formulated with 100 μl of Alhydrogel adjuvant 2% (InvivoGen). Tumour growth was followed daily and measured using calipers. Tumours were collected and measured on day 14. TILs were isolated by treating the tumours with collagenase D (Roch) in 10%FSC containing DMEM for 25 min at 37 °C. Cells were passed through a cell strainer of 100 μm (Corning) and TILs were separated using Ficoll (Sigma-Aldrich). TILs were stained with Fc block, live/dead, anti-CD8 (eBioscience) and p33 tetramers (TCMetrix).

### Statistics

Tumour growth curves were compared by calculating the area-under curve (AUC) and analyzed by One-Way ANOVA (Turkey’s Multiple Comparison Test). Other data has been analyzed and presented using Unpaired Student’s *t* test. GraphPad Prism7 or 8 software was used for the analysis.

## Results

### CuMV_TT_-VLPs demonstrate fast kinetics and constitute an efficient vaccine platform for displaying target peptides/epitopes

**Fig. 1:**
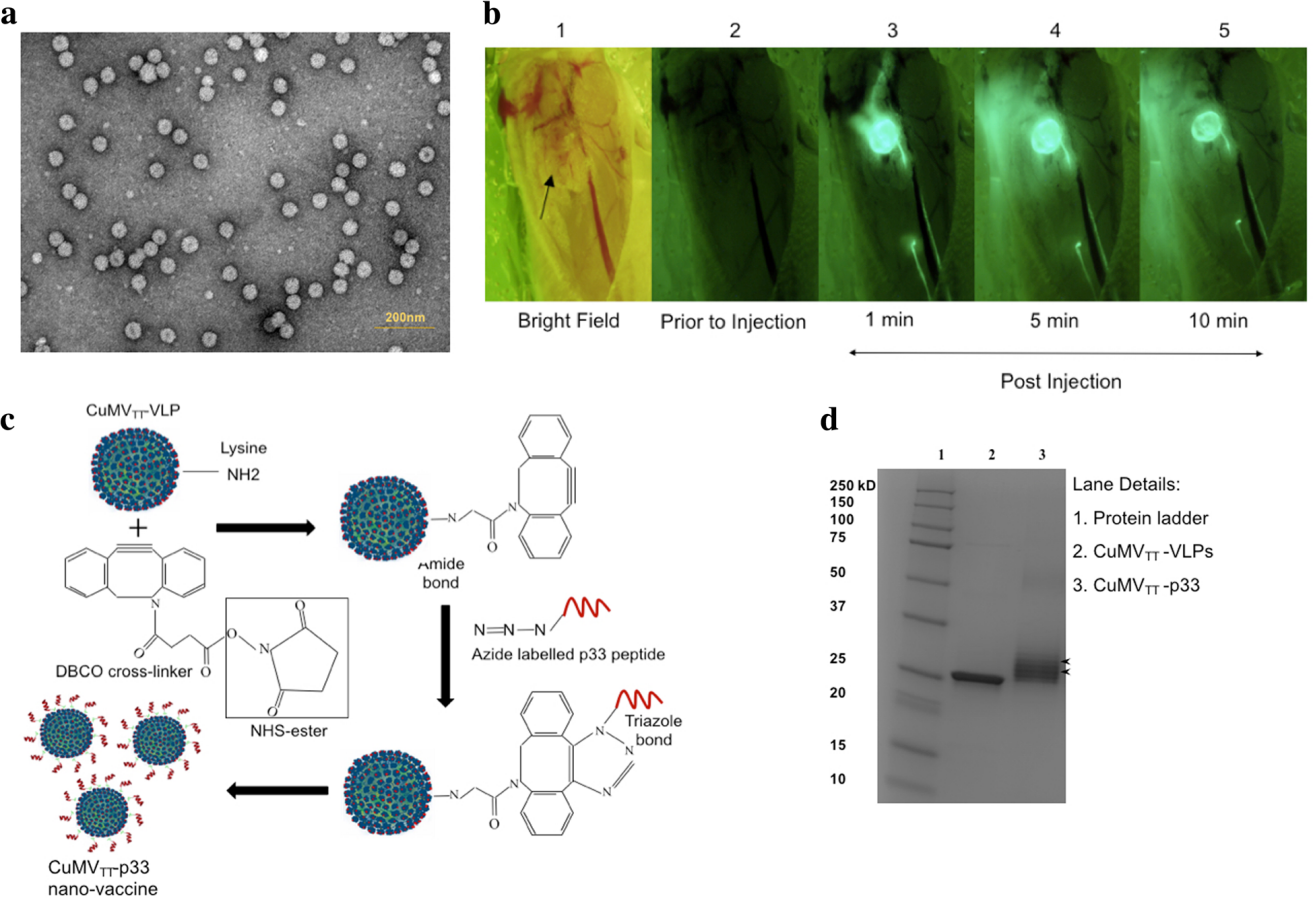
CuMV_TT_-VLPs demonstrate fast kinetics and constitute an efficient vaccine platform for displaying target peptides/epitopes. **a** Electron microscopy imaging of CuMV_TT_-VLPs, (3.5 mg/ml) adsorped on carbon grids and negatively stained with uranyl acetate solution, scale bar 200 nm, CuMV_TT_-VLPs sized ∼30 nm. **b** Stereomicroscopy images of mice popliteal LN following s.c. injection of AF488 CuMV_TT_-VLPs in mice footpad. [1] bright field of the popliteal LN (identified by the arrowhead) [2] fluorescent image prior to injection of AF488 CuMV_TT_-VLPs, [3–5] 1 min, 5 min and 10 min post injection of AF488 CuMV_TT_-VLPs taken with the appropriate fluorescent filters. **c** A sketch illustrating the coupling of p33 epitope to CuMV_TT_-VLPs using Cu-free click chemistry (Dibenzocyclooctyne-*N*-hydroxysuccinimidyl ester (DBCO) cross-linker). DBCO cross-linker reacts with Lys residues on CuMV_TT_-VLPs and incorporates a cyclooctyne moiety. The formed dibenzocyclooctyne will then react with azide-labelled p33 peptide forming a stable triazole linkage without Cu catalyst. **d** SDS-PAGE of CuMV_TT_-p33 nano-vaccine using DBCO “Dibenzocyclooctyne-*N*-hydroxysuccinimidyl ester” cross-linker; arrows indicate CuMV_TT_-VLP monomers and dimers (formed by DBCO cross-linking of 2 monomers) with coupled p33 peptide

In a first step, we produced the engineered CuMV_TT_-VLPs and confirmed their morphology, integrity and nano-size by electron microscopy (Fig. 1a). Next, we studied the draining-kinetics of CuMV_TT_-VLPs utilizing stereomiscroscopic imaging. To this end, CuMV_TT_-VLPs were labelled with the fluorescent dye AF488 and injected s.c. in the footpad of WT C57BL/6 mice. Our results show that the labelled nanoparticles accumulate in the popliteal LN in less than 1 min, demonstrating fast and efficient draining of free 30 nm CuMV_TT_-VLPs (Fig. 1b). To study the efficacy of CuMV_TT_-VLPs as a cytotoxic T cell based nano-vaccine, we have used the H-2D^b^ restricted p33 peptide derived from LCMV as a model antigen. The model peptide was coupled to CuMV_TT_-VLPs using our developed method based on “biorthogonal Cu-free click chemistry” as illustrated in Fig. 1c. The efficiency of the coupling was tested using SDS-PAGE. The additional bands above the VLP monomer show efficient coupling of p33 peptide to CuMV_TT_-VLP monomers (Fig. 1d).

### The micron-sized MCT adjuvant displays depot effect when combined with CuMV_TT_-p33 nano-vaccine

**Fig. 2:**
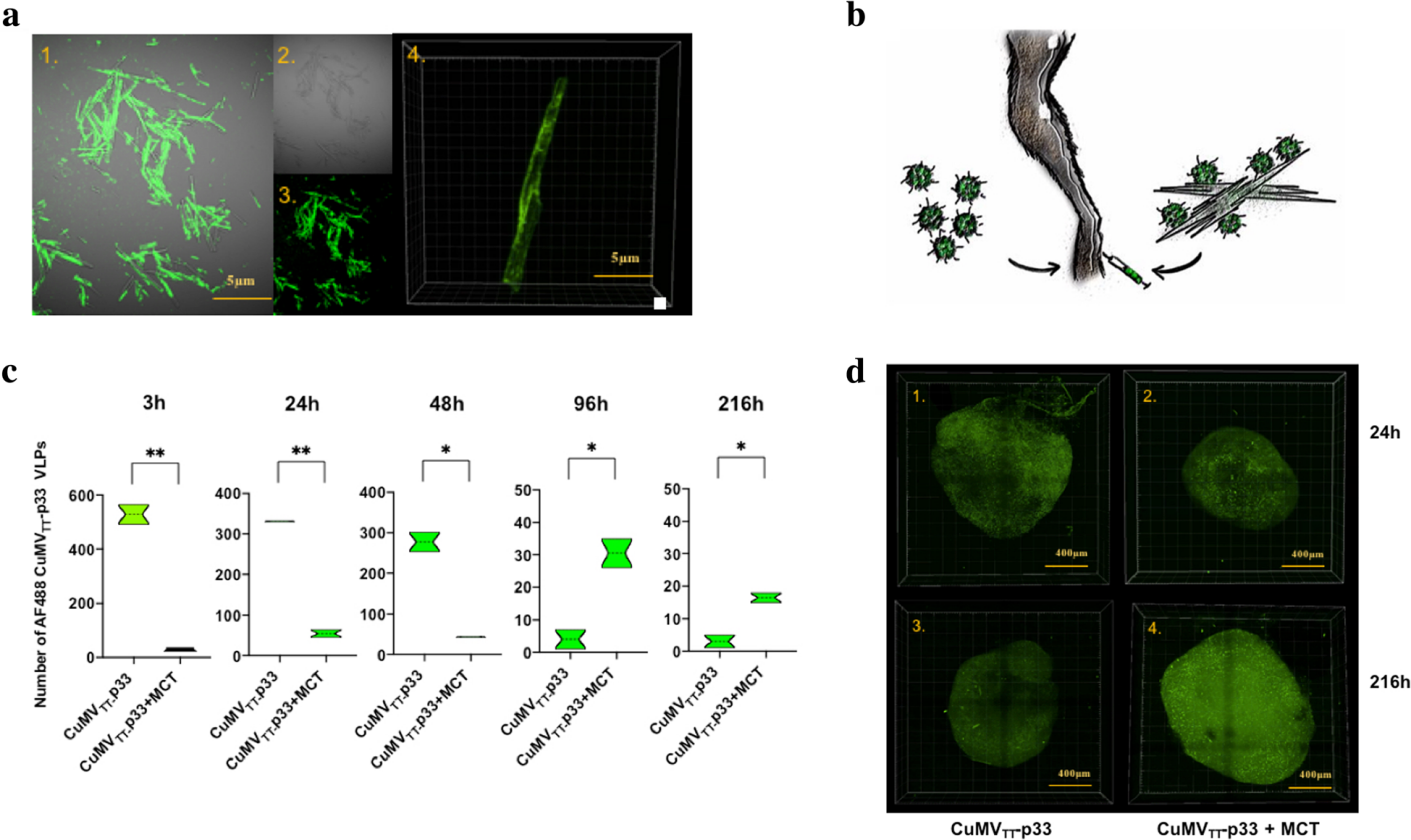
The micron-sized MCT adjuvant displays depot effect when combined with CuMV_TT_-p33 nano-vaccine. **a** Confocal microscopy imaging of AF488 CuMV_TT_-p33 nano-vaccine following formulation with the micron-sized MCT adjuvant, 1) GFP signal of AF488 CuMV_TT_-p33 nano-vaccine 2) bright light field 3) an overlay 4–6 3D images with bright light field 4) an MCT crystal decorated with CuMV_TT_-p33 nano-vaccine particles. **b** A sketch illustrates the two prepared nano-vaccines, the 1st group consists of AF488 CuMV_TT_-p33 nano-vaccine and the 2nd consists of AF488 CuMV_TT_-p33 nano-vaccine formulated with MCT adjuvant. **d** Total number of AF488 CuMV_TT_-p33 nanoparticles in the popliteal LNs collected 3 h, 24 h, 48 h, 96 h and 216 h post-injection of the two prepared nano-vaccine groups in mice footpad. Statistical analysis by unpaired Student’s *t* test. **c** Confocal microscopy images of popliteal LNs 24 h and 216 h post-injection of the two prepared nano-vaccine groups in mice footpad, GFP signal was detected in LNs, whole mount view of z-stacks was acquired. One representative experiment of 3 similar experiments is shown

Microcrystalline tyrosine (MCT) is considered to be a depot-forming adjuvant facilitating the slow but prolonged release of antigens. Formulating the nano-vaccine CuMV_TT_-p33 with the micron-sized MCT adjuvant may therefore enhance the slow release of the nanoparticles displaying the target epitope and extend their exposure to the immune system. To test that, we have first formulated the AF488 CuMV_TT_-p33 nano-vaccine with MCT in vitro to visualize the binding of the nanoparticles to the micron-sized MCT adjuvant by confocal microscopy. The results showed that the labelled nanoparticles bind and decorate the surface of the micron-sized crystals (Fig. 2a). To further study this hypothesis in vivo, we injected the AF488 CuMV_TT_-p33 nano-vaccine (alone or formulated with MCT adjuvant) into the footpad of WT C57BL/6 mice as illustrated in Fig. 2b and collected the popliteal LNs at different time-points 3 h, 24 h, 48 h, 96 h and 216 h to assess the persistence of the labelled nanoparticles by flow cytometry. The results demonstrate that CuMV_TT_-p33 injected in free form disappears from the popliteal LN in ~ 4 days while formulating the nano-vaccine with the micron-sized MCT adjuvant causes slower but prolonged release of the nanoparticles over 9 days (Fig. 2c). These findings were also supported when imaging the popliteal LNs by confocal microscopy (Fig. 2d).

### Formulating CuMV_TT_-p33 nano-vaccine with the micron-sized MCT adjuvant induces significant p33 specific T cell response and enhances cytokines secretion

**Fig. 3:**
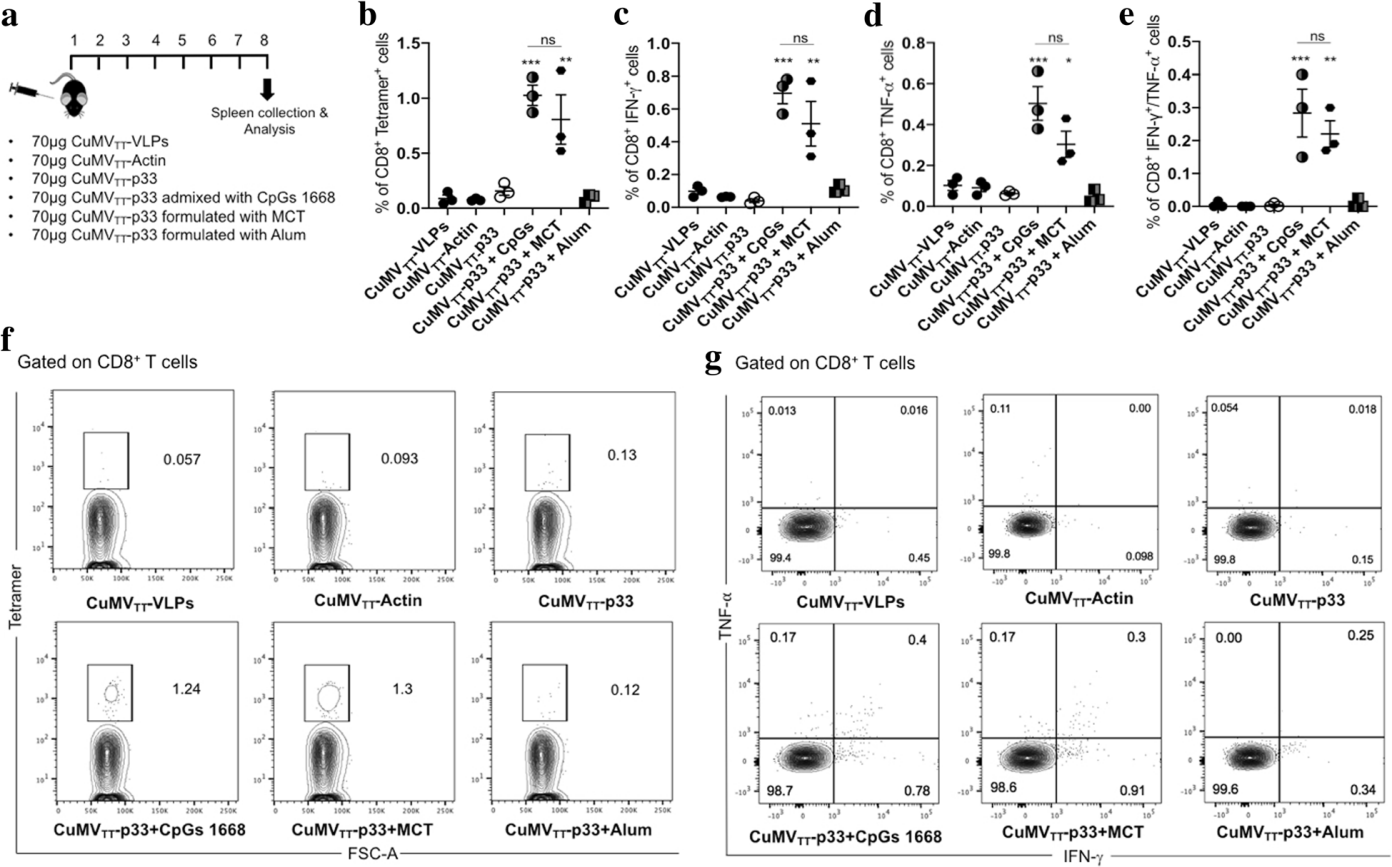
Formulating CuMV_TT_-p33 nano-vaccine with the micron-sized MCT adjuvant induces significant p33 specific T cell response and enhances cytokines secretion. **a** Vaccination scheme for six vaccine groups, CuMV_TT_-VLPs, CuMV_TT_-Actin, CuMV_TT_-p33, CuMV_TT_-p33 admixed with CpG 1668, CuMV_TT_-p33 formulated with MCT and CuMV_TT_-p33 formulated with Alum. **b** Percentage of CD8^+^ Tetramer^+^ CTLs (means ± SEM) in the spleen in each vaccinated group. **c** Percentage of CD8^+^ IFN-γ^+^ secreting cells (means ± SEM) in the spleen in each vaccinated group. **d** Percentage of CD8^+^ TNF-α^+^ cells (means ± SEM) in the spleen in each vaccinated group. **e** Percentage of CD8^+^ IFN-γ^+^/TNF-α^+^ secreting cells (means ± SEM) in the spleen in each vaccinated group. Statistical analysis by Oneway ANOVA (Turkey’s Multiple Comparison Test). **f** Representative flow cytometry dot plots showing the percentage of CD8^+^ Tetramer^+^ CTLs in each vaccinated group. **g** Representative flow cytometry dot plots showing the percentage of CD8^+^ IFN-γ^+^/TNF-α^+^ secreting cells in each vaccinated group. (3 mice per group), one representative of 3 similar experiments is shown

We then tested whether formulating CuMV_TT_-p33 nano-vaccine with MCT would enhance the specific T cell response in vivo. Therefore, six vaccines and formulations were prepared as outlined in Fig. 3a. CpG 1668 and Alum were independently formulated with CuMV_TT_-p33 nano-vaccine to benchmark the potency of the micron-sized MCT adjuvant. Actin coupled to CuMV_TT_-VLPs was used as a non-specific control peptide to show that the obtained response is specific to p33 peptide. The different vaccine formulations were injected once s.c. in WT C57BL/6 mice and spleens were collected seven days later for tetramer and intra-cellular cytokine staining. The H2-D^b^ allele p33 (KAVYNFATM) tetramers have been used to enable direct visualization and quantification of p33 specific T cells. The results showed that admixing CuMV_TT_-p33 nano-vaccine with CpGs 1668 or formulating it with the micron-sized MCT adjuvant induced the highest percentage of p33 specific T cells upon single injection (Fig. 3b and f). We then assessed the cytokine secretion in each group, mainly IFN-γ and TNF-α. The secretion of both cytokines was enhanced when combining the nano-vaccine with CpGs 1668 or MCT adjuvant (Fig. 3c and d). Formulating CuMV_TT_-p33 nano-vaccine with Alum did not enhance the production of p33 specific T cells nor the secretion of IFN-γ or TNF-α. Furthermore, when analyzing the dual secretion of IFN-γ and TNF-α in polyfunctional T cells, a large percentage of the cytokine-producing T cells was found to be polyfunctional in the groups admixed with CpGs 1668 or MCT (Fig. 3e and g).

### Formulating CuMV_TT_-p33 nano-vaccine with the micron-sized MCT adjuvant delays tumour growth and enhances CD8^+^ and p33 specific CTL infiltration into B16F10p33 tumours

**Fig. 4:**
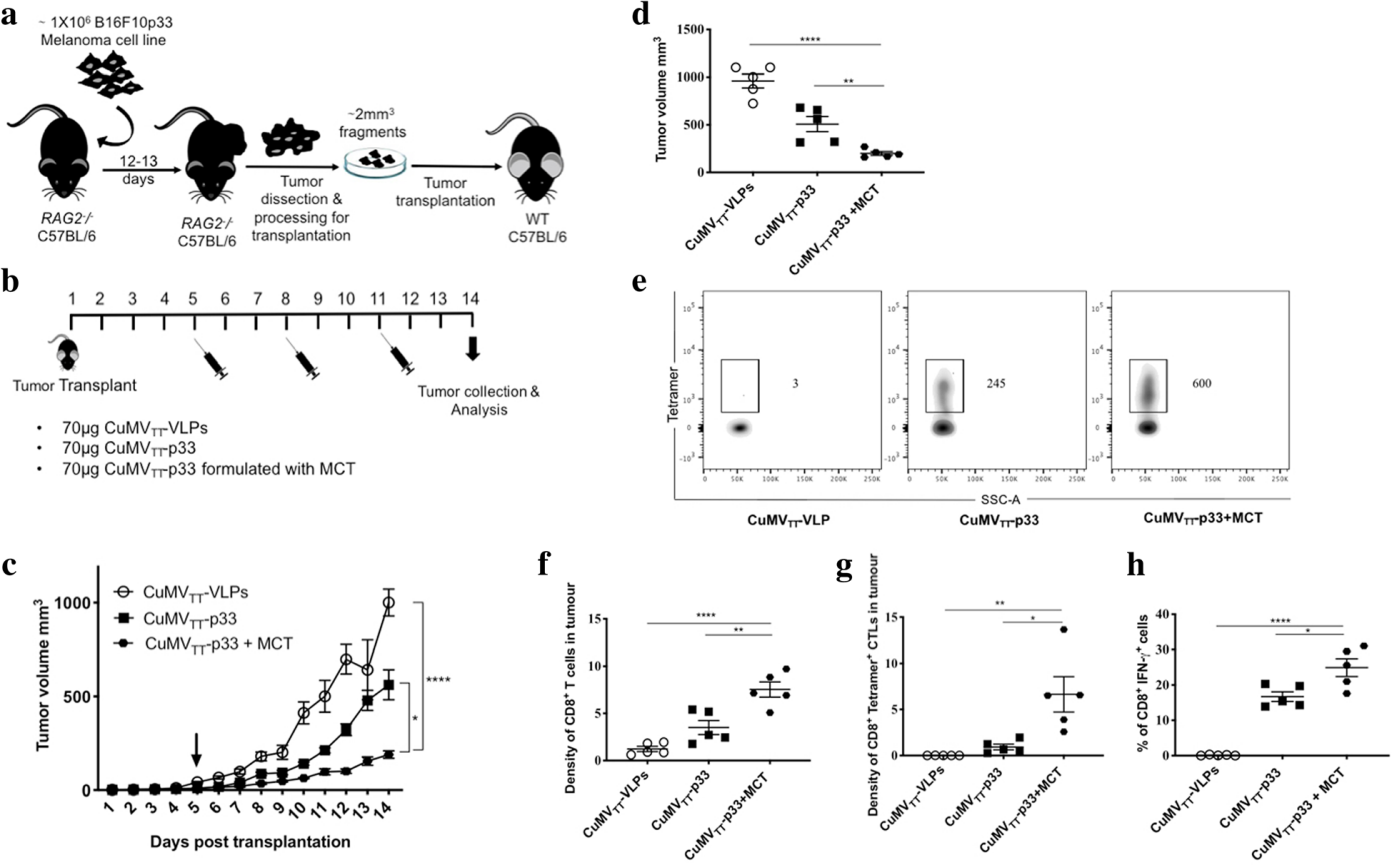
Formulating CuMV_TT_-p33 nano-vaccine with the micron-sized MCT adjuvant causes tumour regression and enhances CD8^+^ and p33 specific CTL infiltration into B16F10p33 tumours. **a** A diagram illustrating the adapted tumour experimental method based on injecting ~ 1 × 10^6^ B16F10p33 melanoma cell line into the flank of *RAG2*^*−/−*^ deficient C57BL/6 mice. Twelve to thirteen days later the growing tumours were collected and processed in ~2mm^3^ for transplantation into the flank of WT C57BL/6. **b** Vaccination scheme for three vaccine groups, CuMV_TT_-VLPs, CuMV_TT_-p33 and CuMV_TT_-p33 formulated with MCT. **c** Tumour growth curve of subcutaneous B16F10p33 melanomas in each vaccinated group, mice were euthanized when the tumour reached ~1000mm^3^, arrows indicate start of treatment. **d** Tumour volume mm^3^ (mean ± SEM) measured at day 14 post tumour collection in each vaccinated group, each dot represents a tumour. **e** Representative flow cytometry dot plots showing the total number of CD8^+^ Tetramer^+^ CTLs in each vaccinated group. **f** Density of CD8^+^ T cells (means ± SEM) in each vaccinated group, “measured by dividing the total number of CD8^+^ cells in TILs by the tumour volume^”^. **g** Density of CD8^+^ Tetramer^+^ CTLs (means ± SEM) in each vaccinated group, “measured by dividing the total number of CD8^+^ Tetramer^+^ CTL by tumour volume^”^. **h** Percentage of CD8^+^ IFN-γ ^+^ secreting cells (means ± SEM) in each vaccinated group. Statistical analysis by Student’s *t* test. (5 mice per group), one representative of 3 similar experiments is shown

In order to test the immunogenicity and efficacy of combining nanoparticles with micron-sized adjuvants in a melanoma model, we have adapted a challenging melanoma murine model based on transplanting ~2mm^3^ of B16F10p33 tumour fragment into the flank of WT C57BL/6 mice. The tumour was allowed to grow for 5 days more following transplantation before the vaccination regimen started (Fig. 4a). Three groups were prepared as illustrated in Fig. 4b, CuMV_TT_-VLPs as a control, CuMV_TT_-p33 nano-vaccine alone and CuMV_TT_-p33 nano-vaccine formulated with MCT. Tumours were collected for analysis 14 days after tumour transplantation as the control group reached the ethically allowed maximal size of ~1000mm^3^. The obtained results revealed that formulating CuMV_TT_-p33 nano-vaccine with the micron-sized MCT could significantly hinder B16F10p33 tumour progression when compared to the control group (*p* < 0.0001) or to the group vaccinated with CuMV_TT_-p33 nano-vaccine alone (*p* 0.0055) (Fig. 4c and d). Tumour-infiltrating lymphocytes (TILs) represent a prognostic factor for effective immune responses especially in melanoma [21, 22]. Therefore, we measured the total number of the infiltrated CD8^+^ T cells and p33 specific CTLs (Fig. 4e) in TILs and calculated the density of these cells in each vaccinated group (number of cells divided by tumour volume). Formulating CuMV_TT_-p33 nano-vaccine with MCT significantly increased the density of total CD8^+^ T cells (*p*. 0.0024) (Fig. 4f) as well as the density of p33 specific CTL measured by tetramers (*p.* 0.0093) (Fig. 4g) in comparison to mice vaccinated with CuMV_TT_-p33 nano-vaccine alone. IFN-γ production was also enhanced when formulating CuMV_TT_-p33 with MCT (Fig. 4h). Thus, formulating the a nano-vaccine in MCT enhanced infiltration by specific T cells as well as anti-tumor protection.

### The micron-sized MCT adjuvant shows comparable activity to B-type CpGs and is superior to alum in driving protection against B16F10p33 melanoma

**Fig. 5:**
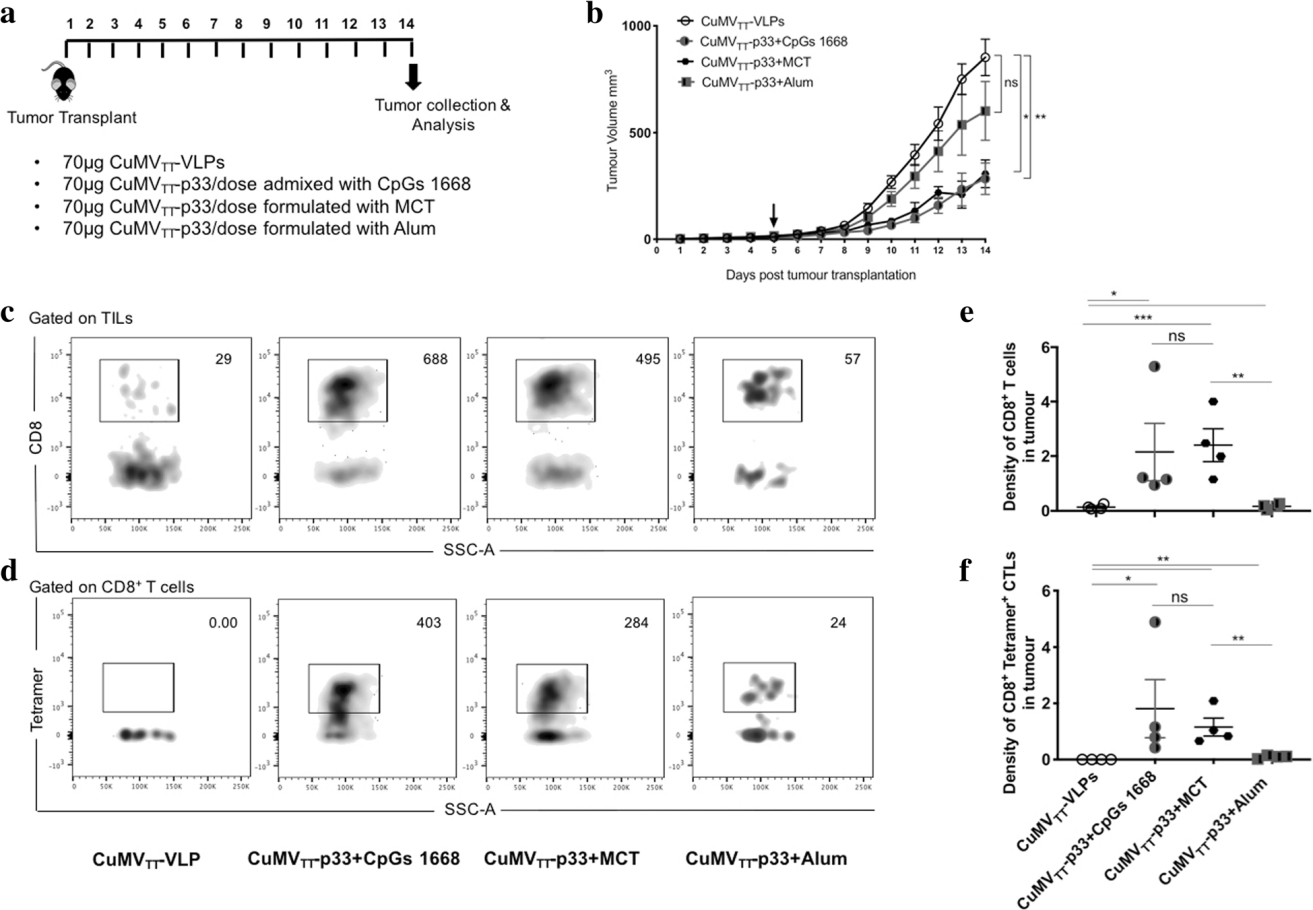
The micron-sized MCT adjuvant shows comparable activity to B type CpGs and is superior to Alum in driving protection against B16F10p33 melanoma. **a** vaccination scheme for four vaccine groups, CuMV_TT_-VLPs, CuMV_TT_-p33 admixed with CpG 1668, CuMV_TT_-p33 formulated with MCT and CuMV_TT_-p33 formulated with Alum. **b** Tumour growth curve of subcutaneous B16F10p33 melanomas in each vaccinated group, mice were euthanized when the tumour reached ~1000mm^3^, arrows indicate start of treatment. **c** Representative flow cytometry dot plots showing the total number of CD8^+^ T cells in each vaccinated group, gated on TILs. **d** Representative flow cytometry dot plots showing the total number of CD8^+^ Tetramer^+^ CTLs in each vaccinated group, gated on CD8^+^ T cells. **e** Density of CD8^+^ T cells (means ± SEM) in each vaccinated group, “measured by dividing the total number of CD8^+^ cells in TILs by the tumour volume^”^. **f** Density of CD8^+^ Tetramer^+^ CTLs (means ± SEM) in each vaccinated group, “measured by dividing the total number of p33 tetramer^+^ CTLs by tumour volume^”^. Statistical analysis by Student’s *t* test. (4 mice per group), one representative of 3 similar experiments is shown

The immunogenicity of the CuMV_TT_-p33 nano-vaccine formulated with the micron-sized MCT adjuvant was then compared to the immune-stimulatory B type CpGs and the widely used adjuvant Alum using the same aggressive B16F10p33 tumour model. Four groups were prepared as shown in Fig. 5a. The results again revealed that formulating CuMV_TT_-p33 nano-vaccine with CpGs 1668 or MCT would significantly (*p* 0.0072, 0.0129 respectively) hinder B16F10p33 tumour progression, which was not the case when formulating CuMV_TT_-p33 with Alum (*p* 0.4188) (Fig. 5b). In a next step, we measured the total number of infiltrated CD8^+^ T cells (Fig. 5c) and p33 specific CTLs (Fig. 5d) in the tumour and calculated the density. There was a general increase in the groups mixed with CpGs 1668 or formulated with MCT or Alum (Fig. 5e and f). Formulating CuMV_TT_-p33 nano-vaccine with the micron-sized MCT adjuvant showed comparable results to CpGs 1668, the gold standard adjuvant in mice. Furthermore, MCT adjuvant was more potent at increasing the infiltration of total CD8^+^ and p33-specific T cells into the tumour microenvironment when compared to formulating the vaccine with Alum.

### The micron-sized MCT adjuvant shows comparable production of cytokines to B type CpGs and is superior to alum in B16F10p33 melanoma

**Fig. 6:**
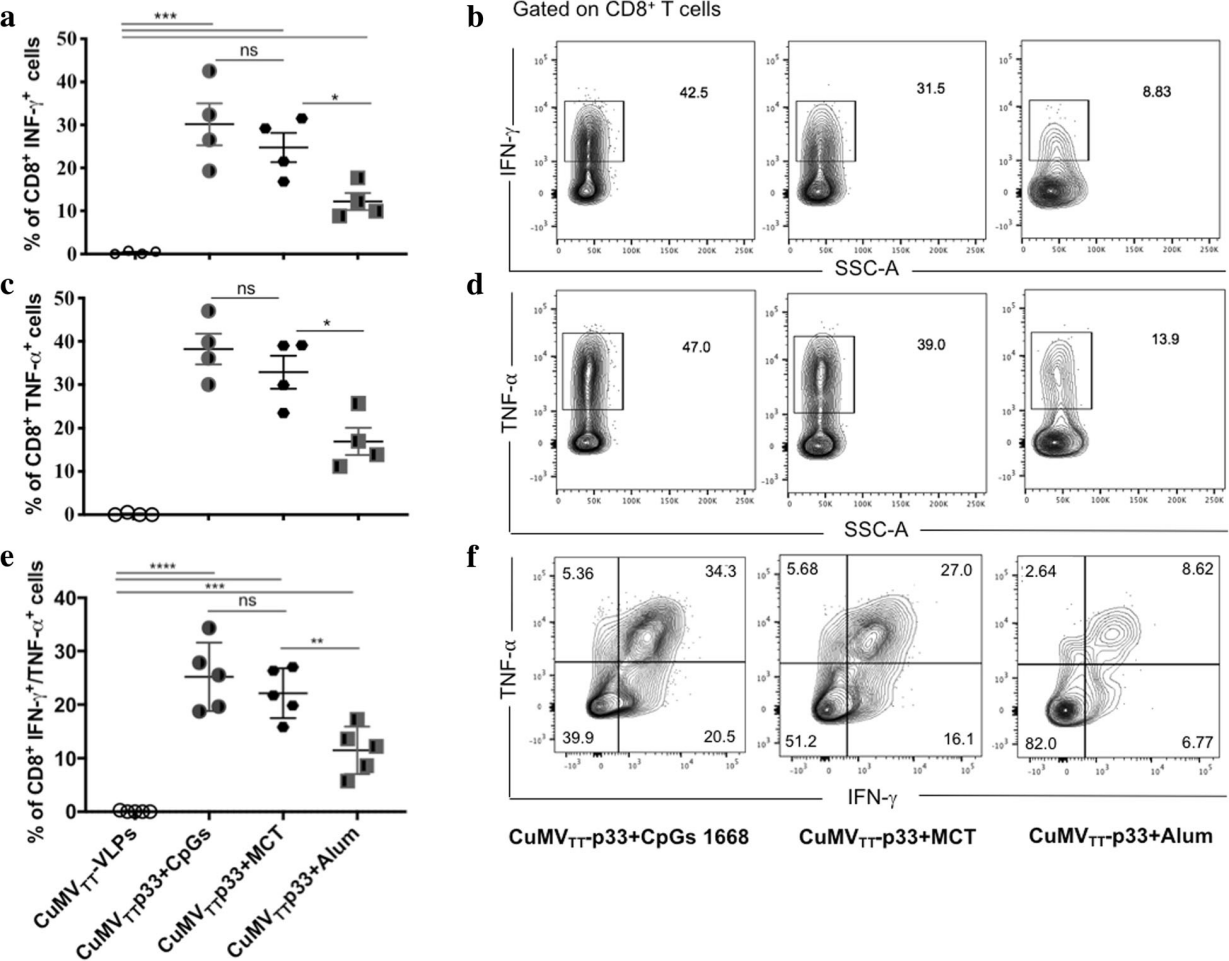
The micron-sized MCT adjuvant shows comparable production of cytokines to B type CpGs and is superior to Alum in B16F10p33 melanoma. **a** Percentage of CD8^+^ IFN-γ ^+^ secreting cells (means ± SEM) in each vaccinated group. **b** Representative flow cytometry dot plots showing the frequency of CD8^+^ IFN-γ^+^ secreting cells in each vaccinated group. **c** Percentage of CD8^+^ TNF-⍺^+^ secreting cells (means ± SEM) in each vaccinated group. **d** Representative flow cytometry dot plots showing the frequency of CD8^+^ TNF-⍺^+^ secreting cells in each vaccinated group. **e** Percentage of dual IFN-γ^+^ and TNF-⍺^+^ secreting cells (means ± SEM) in each vaccinated tumour, gated on CD8^+^ cells in TILs. **f** Representative flow cytometry dot plots showing the frequency of dual IFN-γ^+^ and TNF-⍺^+^ secreting cells in each group, gated on CD8^+^ cells in TILs. Statistical analysis by Student’s *t* test. (4 mice per group), one representative of 3 similar experiments is shown

Production of IFN-γ and TNF-*a* cytokines by TILs was also assessed upon immunization with CuMV_TT_-p33 admixed with B type CpGs or formulated with MCT or Alum. Production of IFN-γ (Fig. 6a and b) and TNF-*a* (Fig. 6c and d) was assessed separately or as dual cytokine production in polyfunational T cells (Fig. 6e and f). There was no significant difference in the production of single IFN-γ, TNF-*a* or dual IFN-γ/TNF cytokines between the groups admixed with CpGs 1668 or formulated with MCT adjuvant (*p* 0.3986, 0.3433 and 0.4120 *respectively*). However, there was a significant difference when comparing the group formulated with MCT to the one formulated with Alum (*p* 0.0179, 0.0187 and 0.006, *respectively*).

## Discussion

In this study, we have developed cucumber mosaic virus-derived nanoparticles genetically fused to the universal T cell epitope of Tetanus toxin (CuMV_TT_-VLPs). These nanoparticles constitute a promising vaccine platform as the incorporated Tetanus toxin epitope can enhance their immunogenicity and the production of robust Ab and CTL responses especially in aging populations [5, 6]. Displaying epitopes on CuMV_TT_-VLP’s exterior surface can be achieved by simple chemical techniques such as the SMPH heterobifunctional cross-linker. Such techniques have shown efficacy and good results in many different clinical settings [23–25]. However, we have lately enhanced the coupling efficacy of epitopes to bacteriophage Qβ-VLP using the biorthogonal Cu-free click chemistry (Mohsen et al., submitted). Here we have shown that such method can also be efficiently used to couple peptides/epitopes to CuMV_TT_-VLPs. Generally, Cu-free click chemistry is a safe, non-toxic coupling method as the azide moiety attached to the target epitope does not react with any of the body’s natural molecules [26–28].

Immunostimulatory adjuvants such as synthetic CpG-oligonucleotides are TLR-agonists and have shown promising therapeutic potential by activating both the innate and adaptive immune system [29–31]. CpGs have also been successfully used clinically to adjuvant cancer vaccines [32, 33]. However, CpGs have some drawbacks including their unfavourable pharmacokinetics and their propensity to cause splenomegaly, at least in mice [34, 35]. It has been shown previously that packaging CpGs into VLPs such as Qβ or HBcAg can improve the pharmacokinetics and dynamics of the DNA oligomers [36]. Nevertheless, it is not always feasible to package VLPs with CpGs as some nanoparticles are unstable and packaging with reassembly processes may be time consuming when targeting translational approaches. Furthermore, TLR-9 agonists have been widely used with VLP-based vaccines to enhance T and B cell responses mostly in mice and more rarely in humans [37]. In mice, TLR-9 is expressed by all DCs while in human it is mainly expressed by pDCs in lymphoid organs but not by conventional DCs [38]. pDCs, however, respond much more efficiently to A type CpGs rather than B type CpGs as only the former induce strong production of type I IFN [39]. Hence, it may be difficult to directly translate findings with B type CpGs from mice to humans. With this respect, it is interesting to note that CuMV_TT_ VLPs naturally package RNA from the *E. coli* expression strain, a ligand for TLR7/8 which is expressed in all human DCs.

Several preclinical and clinical studies have indicated that some of the adjuvants used in licensed products are not optimal for developing effective cancer vaccines. Examples are the commonly used Montanide (Incomplete Freund’s adjuvant) and Alum [40–43]. Such limitations may include the inadequate ability of these adjuvants to induce CTLs [44]. Nevertheless, use of B-type CpGs and Alum in mice is useful where direct comparative adjuvant studies are concerned. Since they are well characterized in mice, both adjuvants are a good benchmark when comparing other new or newly used adjuvants such as MCT.

MCT is a micron-sized adjuvant that forms crystals of about ~ 5 μM that readily adsorb proteins including protein-based nanoparticles such as VLPs. MCT is well known in the world of specific allergy immunotherapy, as it is categorized as a depot excipient in registered subcutaneous immunotherapy products for the treatment of allergies. However, the knowledge about its mechanism of action and its potential in different fields of vaccinology is only expanding now. In a recent study, MCT was shown to be an effective adjuvant in allergen-specific immunotherapy. Protection against IgE-mediated allergic response was achieved in mouse models, independently of inflammasome and TLR signaling in vivo. As has been seen for Alum, the adjuvant activity of MCT was independent of the inflammasome in vivo despite its ability to activate the inflammasome in vitro [11]. In malaria, MCT has been shown to consistently enhance protective IgG responses and more protective IgG subclasses resulting in enhanced protection against malaria. Similar observations were made for influenza vaccine candidates [13, 14, 17].

Here, we have studied the draining kinetics of CuMV_TT_-p33 vaccine alone or formulated with adjuvant MCT. Our results indicate that the free AF488 CuMV_TT_-p33 can drain rapidly into the draining LN but fades away after ~ 4 days. In contrast, the release of AF488 CuMV_TT_-p33 nano-vaccine formulated with MCT was delayed and more consistent over a longer period of time. Previous studies have mostly supported the importance of the depot-forming adjuvants in T cell based vaccine development as they delay the clearance of the vaccine that results in enhancing the generation of effective antigen-specific CTL responses [45, 46].

To study the immunogenicity of the developed CuMV_TT_-p33 nano-vaccine formulated with MCT, the protective capacity of the induced CTL-responses was assessed in an aggressive murine melanoma model consisting of B16F10 cells transfected with H-2D^b^ restricted p33 epitope derived from LCMV. To generate tumors with maximal physiological properties, we have used a challenging tumour model based on transplanting solid tumour fragments rather than single cell suspension. The transplantation of solid tumor fragments allows studying tumor development in the context of already established tumor stroma which is more reflective of the physiological situation. When transplanted as solid fragments, even very immunogenic tumors grow in immunocompetent hosts and the vascularized tumours rapidly grow to lethal size (47–50). The results show that formulating CuMV_TT_-p33 nano-vaccine with MCT was more potent in blocking tumour growth than using the nano-vaccine alone. The protective capacity of the CuMV_TT_-p33 with MCT is therefore strong, as the model used is very challenging. Previous studies have indicated that melanoma tumours exhibiting increased numbers of tumour infiltrating CD8^+^ T cells have better prognosis. TILs have led to a better understating of the interaction between hosts and tumours, mainly because their study allowed better characterization of effective therapeutic responses [22, 47]. TILs isolated from the vaccinated groups have been assessed for the presence of p33 specific CTLs by means of tetramers and intracellular cytokine staining. CuMV_TT_-p33 vaccine formulated with MCT adjuvant enhanced the infiltration of CD8^+^ and p33 specific CTLs into the tumour and the production of IFN-γ. These results indicate that MCT may be a promising cancer adjuvant. When comparing MCT adjuvant to the potent B type CpGs or the widely used Alum, the overall adjuvants activity of MCT was comparable to CpGs and superior to Alum.

It has been previously seen that Alum may increase overall IgG responses at least as good as MCT. In contrast to Alum, however, MCT induced superior IgG2a responses, which is usually associated with T_H_1 responses and/or TLR activity [14]. This may be compatible with the observed ability of MCT to enhance CTL responses, which Alum failed to do. Further work will be required to elucidate the mechanism of this difference, as both Alum and MCT form a depot and may activate the inflammasome pathway. An obvious hypothesis is that Alum induces a T_H_2 driving pathway in addition to the inflammasome or vice versa, MCT may activate a T_H_1 driving pathway. The distinctions portrayed in their respective immunological profiles are also likely to be be partly governed by their inherent formulation/structural characteristics (i.e. particle size, morphology, antigen adsorption etc). In addition to this, it is known that tryptophan or arginine serve as direct immune-modulators, a possibility that has not been extensively studied for tyrosine [48, 49].

Taken together, this study shows that MCT is a potent enhancer of CTL responses and may be viewed as a multi-purpose adjuvant with novel indications. As such, its effectiveness and compatibility in a mix- and match adjuvant systems approach should be further tested in preclinical and human immunotherapy trials. Combination of MCT with nanoparticles appears particularly attractive, as the micron-sized adjuvants will form a local depot at the injection site with concomitant activation of skin-resident antigen-presenting cells. Nanoparticles will be released over time, draining to local LNs for extended time-periods, causing an optimal immune reaction. Thus, the combination of nanoparticles with micron-sized adjuvants may optimally harness the properties of the lymphatic system.


**Additional file 2: Movie S2** showing the popliteal LN 24 h after injecting CuMV_TT_-p33 nano-vaccine in mice footpad. (MP4 19763 kb)



**Additional file 4: Movie S4** showing the popliteal LN 216 h after injecting CuMV_TT_-p33 nano-vaccine in mice footpad. (MP4 30385 kb)



**Additional file 5: Movie S5** showing the popliteal LN 216 h after injecting CuMV_TT_-p33 nano-vaccine formulated with MCT adjuvant in mice footpad. (MP4 34519 kb)


## Additional files

Additional file 1: **Movie S1.** showing AF488 CuMV_TT_-VLPs decorating the surface of microcrystalline tyrosine crystals MCT adjuvant. (MP4 13481 kb)

Additional file 3: **Movie S3.** showing the popliteal LN 24 h after injecting CuMV_TT_-p33 nano-vaccine formulated with MCT adjuvant in mice footpad. (MP4 12060 kb)
