# Correction to: Vaccination with nanoparticles combined with micro-adjuvants protects against cancer

**DOI:** 10.1186/s40425-019-0616-y

**Published:** 2019-05-23

**Authors:** Mona O. Mohsen, Matthew D. Heath, Gustavo Cabral-Miranda, Cyrill Lipp, Andris Zeltins, Marcos Sande, Jens V. Stein, Carsten Riether, Elisa Roesti, Lisha Zha, Paul Engeroff, Aadil El-Turabi, Thomas M. Kundig, Monique Vogel, Murray A. Skinner, Daniel E. Speiser, Alexander Knuth, Matthias F. Kramer, Martin F. Bachmann

**Affiliations:** 1 Jenner Institute, Nuffield Department of Medicine, University of Oxford, Oxford, UK; 2 Department of BioMedical Research, Immunology RIA, Inselspital, University of Bern, Bern, Switzerland; 3 Bencard Adjuvant Systems, Dominion Way, Worthing, UK; 4 Latvian Biomedical Research & Study Centre, Riga, Latvia; 5 Institute of anatomy, University of Bern, Bern, Switzerland; 6 Theodor Kocher Institute, University of Bern, Bern, Switzerland; 7 Department of Medical Oncology, Bern University Hospital, University of Bern, Bern, Switzerland; 8 International Immunology Center, Anhui Agricultural University, Hefei, Anhui China; 9 Department of Dermatology, University of Zurich, Zurich, Switzerland; 10 Department of Oncology, University of Lausanne, Lausanne, Switzerland; 11 National Center for Cancer Care & Research (NCCCR), Doha, State of Qatar

**Correction to: J ImmunoTherapy Cancer**

**https://doi.org/10.1186/s40425-019-0587-z**

Following publication of the original article [1], the authors reported an author’s family name has been erroneously spelled. Paul Engroff should be replaced Paul Engeroff.

Furthermore, there are two errors in the following affiliations: 9) Department of dermatology, University of Zurich, Bern, Switzerland and 10) Department of Oncology, University of Lausanne, Bern,Switzerland should be replaced with 9) Department of dermatology, University of Zurich, Zurich, Switzerland and 10) Department of Oncology, University of Lausanne, Lausanne, Switzerland.

The corrections have been implemented in the original article as well.

The publisher apologized for any inconvenience this might has caused.
